# Inhibitory Effect of* Enterococcus faecium* WB2000 on Volatile Sulfur Compound Production by* Porphyromonas gingivalis*

**DOI:** 10.1155/2016/8241681

**Published:** 2016-10-09

**Authors:** Nao Suzuki, Takuya Higuchi, Masato Nakajima, Akie Fujimoto, Hiromitsu Morita, Masahiro Yoneda, Takashi Hanioka, Takao Hirofuji

**Affiliations:** ^1^Department of Preventive and Public Health Dentistry, Fukuoka Dental College, 2-15-1 Tamura, Sawara-ku, Fukuoka 814-0193, Japan; ^2^Department of General Dentistry, Fukuoka Dental College, 2-15-1 Tamura, Sawara-ku, Fukuoka 814-0193, Japan

## Abstract

Volatile sulfur compounds (VSCs) produced by oral anaerobes are the major compounds responsible for oral malodor.* Enterococcus faecium* WB2000 is recognized as an antiplaque probiotic bacterium. In this study, the effect of* E. faecium *WB2000 on VSC production by* Porphyromonas gingivalis* was evaluated, and the mechanism of inhibition of oral malodor was investigated.* P. gingivalis* ATCC 33277 was cultured in the presence of four lactic acid bacteria, including* E. faecium* WB2000. Subsequently,* P. gingivalis* ATCC 33277, W50, W83, and two clinical isolates were cultured in the presence or absence of* E. faecium* WB2000, and the emission of VSCs from spent culture medium was measured by gas chromatography. The number of* P. gingivalis* ATCC 33277 in mixed culture with* E. faecium* WB2000 decreased at 6 h, and the rate of decrease was higher than that in mixed cultures with the other lactic acid bacteria. The numbers of five* P. gingivalis* strains decreased at similar rates in mixed culture with* E. faecium* WB2000. The concentration of methyl mercaptan was lower in spent culture medium from* P. gingivalis* and* E. faecium* WB2000 cultures compared with that from* P. gingivalis* alone. Therefore,* E. faecium* WB2000 may reduce oral malodor by inhibiting the growth of* P. gingivalis* and neutralizing methyl mercaptan.

## 1. Introduction

Oral malodor is caused mainly by the metabolism of sulfur amino acids by anaerobic bacteria inhabiting the oral cavity [1]. The main compounds responsible for oral malodor are volatile sulfur compounds (VSCs), such as hydrogen sulfide (H_2_S), methyl mercaptan (CH_3_SH), and dimethyl sulfide; these compounds are produced by some periodontopathic bacteria. Indeed,* Porphyromonas gingivalis*,* Treponema denticola*,* Tannerella forsythia*, and* Prevotella intermedia* generate considerable amounts of H_2_S and CH_3_SH [2].

Probiotic bacteria, defined as live microorganisms that benefit the health of the host when administered in adequate amounts (FAO/WHO 2001), are thought to play a role in the maintenance of oral health [3]. Enterococci are facultatively anaerobic, Gram-positive cocci that form a part of the normal flora of the gastrointestinal tract of animals and humans. They are also frequently found in fermented food, such as cheese and meat [4].* Enterococcus faecium* and* Enterococcus faecalis* are the most clinically relevant members of the genus* Enterococcus*. Traditionally, they are regarded as low-grade pathogens but have emerged as important causes of nosocomial infections [5]. Clinical use of* E. faecium* and* E. faecalis* during food fermentation and as probiotics requires a careful safety evaluation [6].

*E. faecium* strains have been reported to inhibit biofilm formation by cariogenic bacteria* in vitro* [7, 8]. In addition, a previous double-blind randomized trial in which the subjects cleaned their teeth using a dentifrice containing* E. faecium* WB2000 or placebo for 4 weeks revealed improvements in salivary flow, salivary buffering capacity, and plaque accumulation [9]. This study aimed to investigate the effect of* E. faecium* WB2000 on VSC production by* P. gingivalis* and the mechanism of inhibition of oral malodor by* E. faecium* WB2000.

## 2. Materials and Methods

### 2.1. Bacterial Strains and Culture Conditions

The bacterial strains used in the study are listed in Table 1.* Enterococcus faecium* WB2000, previously classified as* Streptococcus faecalis* [10], was provided by Wakamoto Pharmaceutical. The selective medium for* P. gingivalis* consisted of Brucella agar (Becton Dickinson, Le Pont de Claix, France) supplemented with 5% horse blood, hemin (5 *μ*g/mL), vitamin K (1 *μ*g/mL), and gentamicin (50 *μ*g/mL). Lactic acid bacteria were cultivated on BL agar (Nissui, Tokyo, Japan). Bacterial strains were cultivated at 37°C anaerobically for 40 h and suspended in sterile physiological saline to an optical density at 560 nm (OD_560_) of 1.0 for* P. gingivalis* and an OD_560_ of 0.02 for lactic acid bacteria.

### 2.2. Cocultivation of* P. gingivalis* and Lactic Acid Bacteria

Bacterial cocultivation was carried out using 100 *μ*L of* P. gingivalis* suspension, 100 *μ*L of lactic acid bacterial suspension, and 10 mL fresh GAM broth (Nissui, Tokyo, Japan) supplemented with 0.7% glucose, hemin (5 *μ*g/mL), and vitamin K (1 *μ*g/mL) at 37°C anaerobically. Viable bacterial counts in the culture medium were determined at 6, 12, 24, and 48 h on the appropriate agar medium at 37°C anaerobically for 48 h.

### 2.3. Measurement of Volatile Sulfur Compounds

The VSC concentration in spent medium from* P. gingivalis* cultured in the presence or absence of* E. faecium* WB2000 was measured after 24 and 48 h. Aliquots (0.2 mL) of spent culture medium were added to 5 mL conical tubes, which were sealed and incubated at room temperature for 5 min. Then, 0.5 mL of the gas phase was collected and measured by gas chromatography (model GC2014, Shimadzu Works, Kyoto, Japan).

## 3. Results

### 3.1. Effect of Lactic Acid Bacteria on the Growth of* P. gingivalis* ATCC 33277

**Figure 1:**
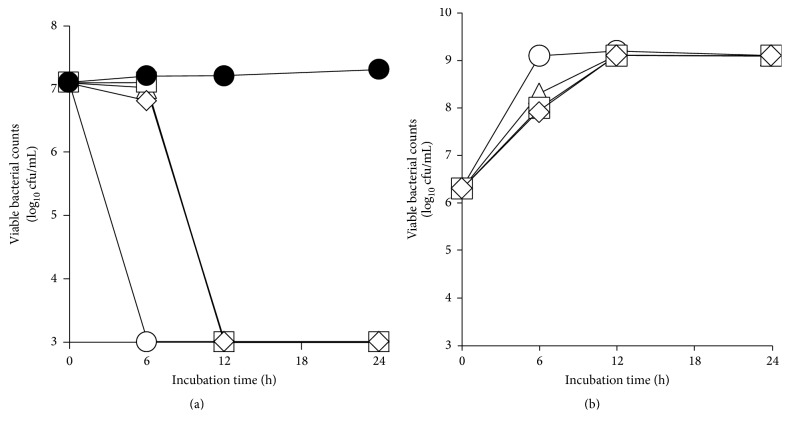
Viable counts of* P. gingivalis* ATCC 33277 in the presence or absence of each of the four lactic acid bacteria (a) and viable counts of lactic acid bacteria in mixed culture with* P. gingivalis* ATCC 33277 (b). ●: number of* P. gingivalis* ATCC 33277 in monoculture, ○: number of* P. gingivalis* ATCC 33277 or* E. faecium* WB2000 in mixed cultures, △: number of* P. gingivalis* ATCC 33277 or* L. salivarius* CIP 103140 in mixed cultures, □: number of* P. gingivalis* ATCC 33277 or* L. reuteri* JCM 1112 in mixed cultures, and ⋄: number of* P. gingivalis* ATCC 33277 or* S. salivarius* JCM 5707 in mixed cultures.

The number of viable* P. gingivalis* ATCC 33277 decreased to less than the detection limit after 6 h in mixed culture with* E. faecium* WB2000 (Figure 1(a)). In mixed cultures with the other three lactic acid bacteria (*S. salivarius* JCM 5707,* L. salivarius *CIP 103140, and* L. reuteri* JCM 1112), the number of viable* P. gingivalis* ATCC 33277 decreased to less than the detection limit at 12 h. In mixed culture with* P. gingivalis* ATCC 33277,* E. faecium* WB2000 grew more rapidly than it did in mixed cultures with the other three lactic acid bacteria, and its growth plateaued at 6 h (Figure 1(b)).

### 3.2. Effect of* E. faecium* WB2000 on the Growth of Various* P. gingivalis* Strains

**Figure 2:**
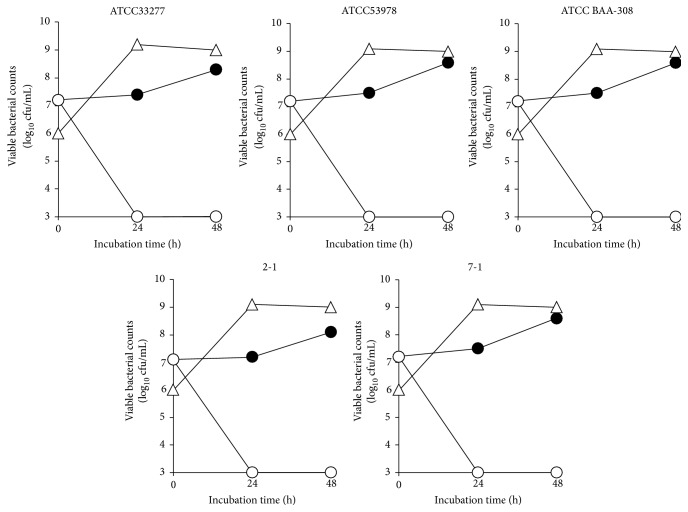
Viable counts of five strains of* P. gingivalis* (ATCC 33277, ATCC 53978, ATCC BAA-308, 2-1, and 7-1) and* E. faecium* WB2000 in mixed cultures. ●: number of* P. gingivalis* in monoculture, ○: number of* P. gingivalis* in mixed cultures with* E. faecium* WB2000, and △: number of* E. faecium* WB2000 in mixed cultures with* P. gingivalis*.

The effect of* E. faecium* WB2000 on the growth of five* P. gingivalis* strains (ATCC 33277, ATCC 53978, ATCC BAA-308, 2-1, and 7-1) was evaluated. The numbers of all* P. gingivalis* strains decreased to less than the detection limit at 24 and 48 h in mixed cultures with* E. faecium* WB2000 (Figure 2). In contrast, the number of* E. faecium* WB2000 reached a plateau at 24 h.

### 3.3. Effect of* E. faecium* WB2000 on VSC Production by* P. gingivalis* Strains

**Figure 3:**
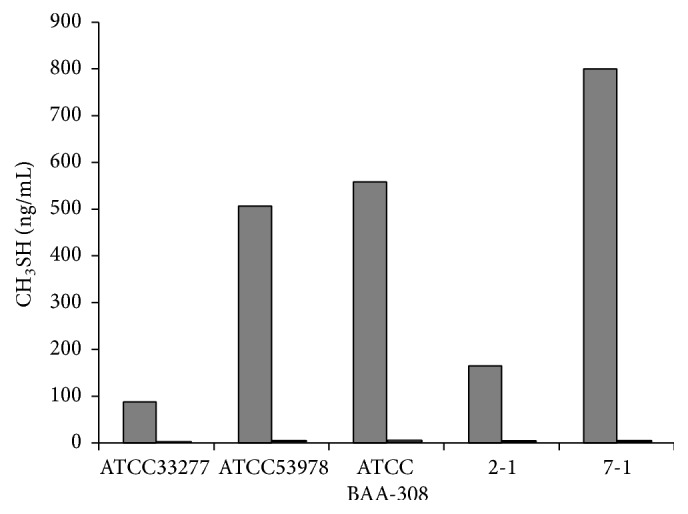
The levels of CH_3_SH in spent medium from* P. gingivalis* cultured for 48 h in the presence or absence of* E. faecium* WB2000 (ng/mL). Grey bars: single culture; white bars: dual culture with* E. faecium* WB2000.

The concentrations of VSCs in spent culture medium were measured by gas chromatography (Table 2). The levels of H_2_S produced by* P. gingivalis* strains were lower than those of CH_3_SH. The concentrations of H_2_S in mixed culture media were higher than that in medium in which only* P. gingivalis* was cultured, with the exception of medium from mixed cultures of two* P. gingivalis* clinical isolates (2-1 and 7-1) and* E. faecium* WB2000 after 48 h. In contrast, the CH_3_SH concentration in mixed culture medium was lower than that in medium from culture of* P. gingivalis* alone, with the exception of medium from mixed cultures of two* P. gingivalis* strains (W50 and 2-1) and* E. faecium* WB2000 after 24 h. The levels of CH_3_SH in spent medium from* P. gingivalis* cultured for 48 h in the presence or absence of* E. faecium* WB2000 are shown in Figure 3. Although CH_3_SH production by* P. gingivalis* was strain dependent, the CH_3_SH concentration was markedly lower in spent medium from mixed cultures of all* P. gingivalis* strains and* E. faecium* WB2000 than in medium from culture of the latter microorganism only.

## 4. Discussion

The hypothetical mechanisms of probiotic action in the oral cavity are (1) involvement in binding of oral microorganisms to proteins, (2) effects on plaque formation and its complex ecosystem by competing and interfering with interbacterial attachment, (3) involvement in the metabolism of substrates, and (4) production of compounds that inhibit oral bacteria [11].* E. faecium* has been reported to inhibit biofilm formation by cariogenic bacteria [7, 8]. Kumada et al. [8] reported a protein that inhibited biofilm formation by streptococci. In addition, our previous study suggested that* E. faecium* inhibits the growth of some mutans streptococci [7]. In this study,* E. faecium* WB2000 inhibited the growth of, as well as reduced CH_3_SH production by,* P. gingivalis*. A study on* L. salivarius* TI 2711 reported that a low pH (≤6.0) and the presence of lactic acid (40–50 mmol/L) induced* P. gingivalis* death [12]. The pH of* E. faecium* WB2000 culture medium was 4.4 after 24 h of incubation (data not shown). The growth rate of* E. faecium* WB2000 was higher than that of the other lactic acid bacteria, suggesting that this organism rapidly inhibited the growth of* P. gingivalis*.

*P. gingivalis* strains produced CH_3_SH and a low level of H_2_S, as reported previously [13].* E. faecium* WB2000 suppressed CH_3_SH production by* P. gingivalis* but did not inhibit production of H_2_S in the current study. Some probiotic bacteria produce VSCs in the presence of cysteine or methionine [14]. The GAM broth used in the current study contains cysteine, and thus* E. faecium* WB2000 might have produced H_2_S.* Streptococcus thermophilus* inhibited the growth and H_2_S, CH_3_SH, and CH_3_SCH_3_ production of* P. gingivalis* [13]. To determine whether* E. faecium* WB2000 specifically inhibits production of CH_3_SH, different media and culture conditions should be used. Furthermore, future studies are needed to evaluate other* P. gingivalis* strains producing high levels of H_2_S.

In addition to* E. faecium* and* S. thermophilus*, other organisms have been reported to inhibit oral malodor.* Streptococcus salivarius* K12 suppressed the growth of* Solobacterium moorei*, which produces H_2_S and is involved in halitosis [15, 16].* Lactobacillus salivarius* WB21 and* L. reuteri* have been reported to inhibit oral malodor in clinical trials [17–19].* L. salivarius* WB21 reduced the number of* Fusobacterium nucleatum* and ubiquitous bacteria in saliva [19].

*E. faecium* WB2000 has been used in traditional Japanese medicine (Strong Wakamoto®) to treat gastrointestinal discomfort, and its effect on dry eye was reported recently [20]. Moreover, the effect of dentifrice containing* E. faecium* WB2000 on plaque control has been investigated [9]. The findings of this study suggest that* E. faecium* WB2000 may reduce oral malodor by inhibiting the growth of* P. gingivalis* and neutralizing CH_3_SH. The effect of* E. faecium* WB2000 on oral malodor will be investigated in a future clinical trial involving the use of dentifrice.

## Figures and Tables

**Table 1:** Bacterial strains used in the study.

Species	Strain
*Porphyromonas gingivalis*	ATCC 33277
	ATCC 53978 (W50)
	ATCC BAA-308 (W83)
	2-1 (clinical isolate)
	7-1 (clinical isolate)
*Enterococcus faecium*	WB2000
*Lactobacillus salivarius*	CIP 103140
*Lactobacillus reuteri*	JCM 1112
*Streptococcus salivarius*	JCM 5707

**Table 2:** The hydrogen sulfide and methyl mercaptan concentrations in spent culture medium (ng/mL).

Pg strain	Culture medium	Hydrogen sulfide		Methyl mercaptan	
		24 h	48 h	24 h	48 h
ATCC 33277	Pg	0.45	0.86	11.45	87.34
	Pg + Ef WB2000	0.94	1.49	2.38	1.53
ATCC 53978 (W50)	Pg	0.87	2.20	3.40	507.27
	Pg + Ef WB2000	1.44	2.91	4.36	2.92
ATCC BAA-308 (W83)	Pg	0.68	2.46	62.53	558.22
	Pg + Ef WB2000	1.75	2.83	6.61	4.32
2-1	Pg	0.43	2.18	1.70	164.44
	Pg + Ef WB2000	1.11	2.03	3.26	2.81
7-1	Pg	0.86	9.24	58.18	799.95
	Pg + Ef WB2000	2.06	2.44	6.03	4.05

Pg: *Porphyromonas gingivalis*; Ef: *Enterococcus faecium*.
